# Materials and Technology Selection for Construction Projects Supported with the Use of Artificial Intelligence

**DOI:** 10.3390/ma15041282

**Published:** 2022-02-09

**Authors:** Jerzy Rosłon

**Affiliations:** Civil Engineering Faculty, Warsaw University of Technology, Armii Ludowej 16, 00-637 Warszawa, Poland; j.roslon@il.pw.edu.pl

**Keywords:** materials, optimization, value engineering, metaheuristic, artificial intelligence

## Abstract

The choice of material solutions and the appropriate technology for the execution of works have a significant impact on the success of construction projects. The earlier in the investment cycle of a project, the greater the possibility of improving the project’s success indicators. The currently used planning methods assume late integration of schedules with material and technological solutions. This limits the possibility of optimizing construction projects. The author proposed a new approach. The new method is based on the value engineering principles. The article presents a computational model supported by a case study—construction of an office building. Thanks to the use of artificial intelligence and metaheuristic algorithms, the economic results of construction projects have improved. This new method can help construction managers select materials and technologies in a way that will improve project parameters.

## 1. Introduction

The construction projects are characterized by a high level of complexity, a long investment life cycle, and high costs. It results in the necessity to implement special diligence and analysis during their planning [1,2,3,4,5,6]. Choosing both the right material solutions and the appropriate technology for the execution of works has a significant impact on the success of construction projects [7,8,9,10]. According to market research, material costs correspond to most of the construction costs [11,12,13]. What’s more, the selection of appropriate materials translates into many other parameters that are crucial for the projects, including structural safety, fire safety, safety of usage, acoustical comfort, visual comfort, hygrothermal comfort, serviceability, durability, sustainability, and energy conservation [14,15].

However, the choice of materials cannot be made excluding aspects related to specificity of market conditions and the construction industry (including billing systems or contract terms). Restrictions, such as construction duration (deadlines), technological and organizational dependencies, and resources should also be considered while planning construction projects [16].

When building decision-making models, one should also properly select parameters that measure the potential success of projects. According to practitioners and theorists, the success of projects is based on aspects related to cost, time, and quality (meeting the requirements) [17,18,19]. From the contractors’ point of view, the high level of customer satisfaction has a positive effect on winning new contracts, and significantly reduces the probability of being involved in harmful disputes and court hearings and incurring additional costs. If the investor decides to implement the project on their own, it is in their own interest to meet the requirements [20,21,22].

Current value management practices are carried out in the conceptual phase and in the planning phase of project implementation. Meanwhile, the optimization of schedules and related indicators is carried out much later. This oversight causes potential losses in the process of maximizing the value of construction projects in reference to its key economic parameters (net present value, cash flow [14,16,18]), existing conditions, and technological and organizational limitations.

Some of the problems related to the design of the construction process and the choice of materials are attempted to be solved by introducing data-based technologies that work with AI, i.e., novel BIM-based technologies and methodologies working with AI-based systems [1,23,24,25,26,27]. Modern solutions are proposed based on Digital Twins, Extended Reality (XR), Virtual Reality (AR), Augmented Reality, Mixed Reality (MR), Laser Scanners, Drones, etc. Such a comprehensive approach to the topic of construction planning and management results in the creation of complex project models. Despite great advances in IT, the practical construction problems are NP-hard (non-deterministic polynomial time hard) [14,18]. Currently, it is indicated that metaheuristic algorithms are the best way to deal with such problems. However, even they are not guaranteed to find the best solutions [22,28,29]. Therefore, there is a need for ways to support them.

This article presents a concept of solving the above problems. Section 2 describes the value engineering methodology, which is one of the foundations of the new approach. Then, an innovative proprietary optimization model for construction projects was presented. It also describes an innovative way to optimize construction schedules using artificial intelligence.

Section 3 provides a thorough case study showing the benefits of the new method. The example is based on the construction of a modern office building. It deals with the selection of the right materials and the technology of building the facility. This section also summarizes the results of additional studies.

The article ends with a discussion and conclusions. The conclusions drawn from the research were described and further directions of research were indicated.

## 2. Materials and Methods

### 2.1. Value Engineering

Value Engineering (VE) is a methodology or set of principles for defining, maximizing, and achieving the best value of goods, services, or products [14]. The VE term is often used as a synonym of Value Management (VM) [30,31]. VE ensures that the good meets the investor’s needs, especially in terms of materials, costs, and quality. Numerous sources [14,15] underline that the benefits of using VM are especially important during the initial stages of projects. VE/VM is described in detail in many publications [14,15,30,31,32,33].

To use VE, it is important to properly quantify and measure values (functions). The most common practice is to analyze each function/object/element and determine its actual cost of implementation (including principles of sustainable development). Usually, the experts (performing VE) distinguish basic and additional functions influencing the value of an investigated object. They need to analyze all the functions of the element to assess its actual value. For example, when considering material solutions, the primary function of a granite floor is that of a pedestrian walkway. The same role can be played by concrete slabs, the cost of which is lower. However, a granite floor also performs other, additional functions, among which aesthetics (a subjectively measurable function) may play an important role. Granite is also more durable than its concrete counterpart due to its greater abrasion resistance (objectively measurable function) [15].

The author already presented a proprietary approach to the VE in which value (V) was defined as a weighted sum of assessments of the fulfillment of individual functions and aspects related to sustainable development [14]. The approach is based on the value profile tables that use the value creation factors proposed by renowned organizations, such as The International Council for Building (CIB—Conseil International du Bâtiment), the International Organization for Standardization (ISO), the United Nations (UN), and the European Economic Community (EEC).

In the aforementioned approach, individual criteria/factors of creating value get assigned weights, depending on the preferences of a decisionmaker. The results are normalized so that the sum of the weights is equal to 1. As a result, a vector ***Q*** of weights of individual factors for creating the value is created (Equation (1)) [14]:

(1) $$Q=\left[q_{j}\right]\;\overset{n}{\underset{j=1}{\sum }}q_{j}=1\;.$$

Then, individual variants are being assessed in terms of all included criteria (only the ones with scores higher than 0). As a result, the evaluation matrix ***P*** is created. In the next step, variant assessments under individual criteria are being standardized. The elements of the normalized matrix $\bar{P}$ are calculated according to the Equation (2) [14]:

(2) $${\bar{p}}_{ij}=\frac{p_{ij}\;}{\sqrt{\sum_{i=1}^{m}p_{ij}^{2}}}\;i=\bar{1,m}\;,\;j=\bar{1,n}\;,$$

where *n* is the number of value creation factors (criteria), and *m* is the number of assessed variants.

In the next step of the procedure, a normalized ***V*** rating matrix is calculated considering the importance of individual criteria. The elements of the normalized matrix ***V*** are calculated as follows [14]:

(3) $$V_{ij}={\bar{p}}_{ij}\cdot q_{j}\;i=\bar{1,m}\;,\;j=\bar{1,n}\;.$$

The sum of the matrix components in the rows corresponding to the variants is the result of $V_{i}$, which is the score of the individual variants in terms of value creation factors:

(4) $$V_{i}=\overset{n}{\underset{j=1}{\sum }}V_{ij}\;i=\bar{1,m}\;,\;j=\bar{1,n}\;.$$

The results are subject to linear-maximum standardization; thus, we obtain the ***V*** values for all variants of all activities in the schedule. The procedure is presented in detail in [14] and the case study—Section 3 of this paper.

### 2.2. Optimization Model

As already mentioned in the introduction, for the project to be successful, an appropriate analysis must be performed. The author believes that many factors contributing to the success of the project should be analyzed simultaneously, including the specificity of the market and the construction industry, the way of settling works, potential contract conditions, contract terms, technological and organizational dependencies, the life cycle of the facility, the materials used, etc. The author has already presented the appropriate model in the previous work [14]. In this article, the model has been further developed to meet the needs of construction companies. The model uses tested and recommended methods of assessing construction projects: Net Present Value (*NPV*) minimizing monthly cash flows (*CF*) [4,16,18].

Let $R^{\rho }$ and $R^{\nu }$ be sets $r^{\rho }$ and $r^{\nu }$, respectively, of renewable and non-renewable resource types. Their availability: $a_{k}^{\rho }$, $k\in R^{\rho }$ and $a_{l}^{\nu }$, $l\in R^{\nu }$. Each activity *j* consumes $r_{jkt}^{\rho }$ renewable resources and $r_{jlt}^{\nu }$ non-renewable resources during day *t*.

$M_{j\;}$ different modes (variants, for example, use of alternative materials or technology) are introduced in which the activity *j*, $m\in M_{j}=\left\{1,\ldots ,\left|M_{j}\right|\right\}$ can be performed. The duration of action *j* performed in the $m_{j}$ mode is equal to $d_{jm}$. Each of the *m* variants requires $r_{jmk}^{\rho }$ renewable and $r_{jml}^{\nu }$ non-renewable resources. Such notation is characteristic for the MRCPS (Multi-Mode Resource—Constrained Project Scheduling Problem) problems [34,35,36]. It also includes binary variable $x_{jmt}$, taking the value 1, if the activity *j* performed in the mode $m\in M_{j}=\left\{1,\ldots ,\left|M_{j}\right|\right\}\;$ is finished at the end of the period of time *t*. Otherwise $x_{jmt}=0$. $EF_{j}$ and $LF_{j}\;$ are respectively the earliest (early) and late dates for completing the activity *j*.

The new, improved objective function (*O_F_*) aims to maximize parameters such as Net Present Value (*NPV*) and usage/functional value (*V*) while minimizing monthly cash flows (*CF*).

(5) $$\max O_{F}:\;O_{F}=\left(\overset{H+\Delta }{\underset{h=1}{\sum }}\frac{P_{h}-IC_{h}}{{\left(1+\alpha \right)}^{h\;TI}}-\overset{H}{\underset{h=1}{\sum }}\overset{\left|M_{j}\right|}{\underset{m=1}{\sum }}\overset{n}{\underset{j=1}{\sum }}\overset{\min\left\{t+d_{jm}-1,LF_{j}\right\}}{\underset{q=\max\left\{t,EF_{j}\right\}}{\sum }}\frac{CF_{jm}}{d_{jm}\;{\left(1+\alpha \right)}^{t}}\;x_{jmq}\right)\;w_{1}\;+\left(\overset{\left|M_{j}\right|}{\underset{m=1}{\sum }}\overset{n}{\underset{j=1}{\sum }}\overset{LF_{j}}{\underset{t=EF_{j}}{\sum }}\frac{f_{jm}\;x_{jmt}}{J}\;\right)\;w_{2}\;-\left(\underset{t}{\max}\left\{\overset{\left|M_{j}\right|}{\underset{m=1}{\sum }}\overset{n}{\underset{j=1}{\sum }}\overset{\min\left\{t+d_{jm}-1,LF_{j}\right\}}{\underset{q=\max\left\{t,EF_{j}\right\}}{\sum }}\frac{CF_{jm}}{d_{jm}\;{\left(1+\alpha \right)}^{t}}\;x_{jmq}\right\}\right)\;w_{3},\;H=\left\lceil \frac{LF_{j}}{TI}\right\rceil ,\;t=1,\;\ldots ,\;H\;$$

(6) $$\overset{\left|M_{j}\right|}{\underset{m=1}{\sum }}\overset{LF_{j}}{\underset{t=EF_{j}}{\sum }}x_{jmt}=1,\;j=0,\;\ldots ,n+1$$

(7) $$\overset{\left|M_{j}\right|}{\underset{m=1}{\sum }}\overset{LF_{i}}{\underset{t=EF_{i}}{\sum }}t\;x_{imt}\leq \overset{\left|M_{j}\right|}{\underset{m=1}{\sum }}\overset{LF_{j}}{\underset{t=EF_{j}}{\sum }}x_{jmt}\;\left(t-d_{jm}\right),\;\forall \left(i,j\right)\in P$$

(8) $$\overset{n}{\underset{j=1}{\sum }}\overset{\left|M_{j}\right|}{\underset{m=1}{\sum }}\overset{\min\left\{t+d_{jm}-1,LF_{j}\right\}}{\underset{q=\max\left\{t,EF_{j}\right\}}{\sum }}r_{jmk}^{\rho }\;x_{jmq}\leq a_{k}^{\rho },\;k=1,\ldots ,\;r^{\rho }\;,\;t=1,\;\ldots ,\;H$$

(9) $$\overset{n}{\underset{j=1}{\sum }}\overset{\left|M_{j}\right|}{\underset{m=1}{\sum }}\overset{LF_{j}}{\underset{t=EF_{j}}{\sum }}r_{jml}^{\nu }\;x_{jmt}\leq a_{l}^{\nu },\;l=1,\ldots ,\;r^{\nu }$$

(10) $$\overset{LF_{n+1}}{\underset{t=EF_{j}}{\sum }}t\;x_{n+1,m,t}\leq D\;,\;j=0,\;\ldots ,n+1$$

(11) $$x_{jmt}\in \left\{0,1\right\},\;j=0,\ldots ,\;n+1,\;m\in M_{j},\;t=EF_{j},\ldots ,\;LF_{j}$$

where:

- $P_{h}$ are profits for the period ending on *h*, *h* = 1, 2, …, *H*;
- $IC_{h}$ are indirect costs for the period ending on *h*, *h* = 1, 2, …, H;
- *TI* is a known time interval, and in the analyzed model it corresponds to one working month and is expressed in days;
- Δ is a variable for modelling payment delays, where payment delay is ε [working days], Δ=⌈ε/TI⌉;
- $CF_{jm}\;$ is cash flow of activity j performed in mode m;
- α is an interest rate;
- $f_{jm}\;$ is the assessment of the VM functions of activity j performed in mode m;
- $w_{i}$ is a weight of individual parts of the optimization objective function subject to equation $\sum_{1}^{n}w_{i}=1$;
- D is a deadline for completion of construction.

Equation (6) ensures that each activity is performed only once and in only one of the possible modes. (7) models the relations between tasks. The constraints for renewable (8) and non-renewable (9) resources can also be used to model doubly constrained resources. Equation (10) models a deadline for construction completion while constraint (11) is responsible for modeling binary decision variables.

The conceptual notation of the objective function used for computer modeling is similar to the one presented in [14], however, improvements were made to involve the importance of the CF parameter:

(12) $$O_{F}=w_{1}\cdot NPV_{r}+w_{2}\cdot V_{r}-w_{3}\cdot CF_{r}-o_{1}\cdot R-o_{2}\cdot dur$$

where:

- ($w_{1}\cdot NPV_{r}+w_{2}\cdot V_{r})$ is an objective part of the function,
- ($-w_{3}\cdot CF_{r}-o_{1}\cdot R-o_{2}\cdot dur)$ are restrictions (penalties), $w_{i}$ are the weights of individual parts of the objective function subject to optimization,
- $o_{i}$ are the weights of individual parts of the objective function responsible for constraints (penalties).

The sum of $w_{i}$ is equal to 1, while $o_{i}$ values are significantly greater than those of the first part of the objective function (goal), so that failure to meet any of the constraints results in the disqualification of a given solution.

*NPV_r_* is the objective function component responsible for the optimization of the relative NPV value [14]:

(13) $$NPV_{r}=\frac{NPV-NPV_{min}}{NPV_{max}-NPV_{min}}\;,$$

where:

- NPV  is the NPV value for the currently examined case,
- $NPV_{max}\;$ is the maximum NPV value found for the unconstrained version of the project,
- $NPV_{min}\;$ is the minimal NPV value found for the unconstrained version of the project.

*V_r_* is a component of the objective function that corresponds with the score obtained by a given solution in terms of VM principles [14]:

(14) $$V_{r}=\frac{V-V_{min}}{V_{max}-V_{min}}\;,$$

where:

- V  is the value rating for the currently studied case,
- $V_{max}\;$ is the maximum value rating found for the unconstrained version of the tested example,
- $V_{min}\;$ is the minimum value grade found for the unconstrained version of the tested example.

*CF_r_* is the objective function component responsible for the optimization of the relative CF value:

(15) $$CF_{r}=\frac{CF-CF_{min}}{CF_{max}-CF_{min}}\;,$$

where:

- CF  is the CF value for the currently examined case,
- $CF_{max}\;$ is the maximum CF value found for the unconstrained version of the project,
- $CF_{min}\;$ is the minimal CF value found for the unconstrained version of the project.

R is a binary variable responsible for meeting the condition of not exceeding the maximum availability of resources (e.g., workers, machinery, materials) [14].

(16) $$R=\left\{\begin{array}{c} 1\;if\;condition\;\left(8\;or\;9\right)\;is\;not\;met\\ 0\;in\;other\;cases \end{array}\right.$$

*dur* is a binary variable responsible for meeting the condition of not exceeding the contractual construction date [14].

(17) $$dur=\left\{\;\begin{array}{c} 1\;if\;condition\;\left(10\right)\;is\;not\;met\;\\ 0\;in\;other\;cases \end{array}\right.$$

Other aspects and elements of the model presented in [14] remain unchanged.

### 2.3. Optimization Procedure Supported by AI

**Figure 1:**
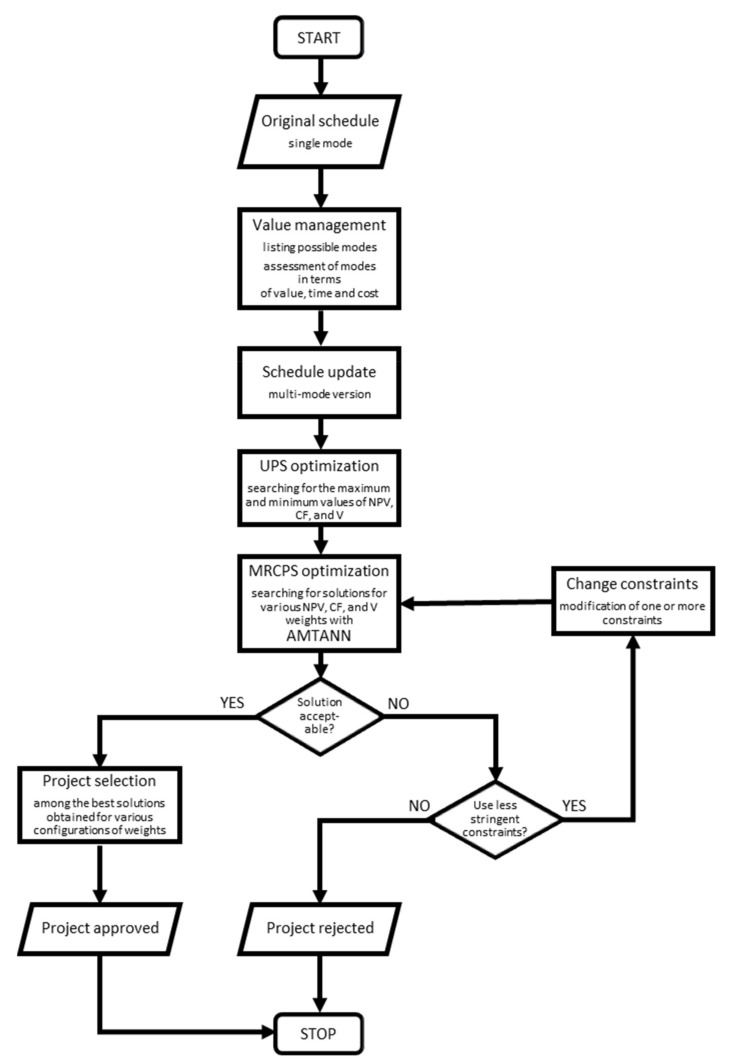
Block diagram of the proposed algorithm—modified on a base of [14].

**Figure 2:**
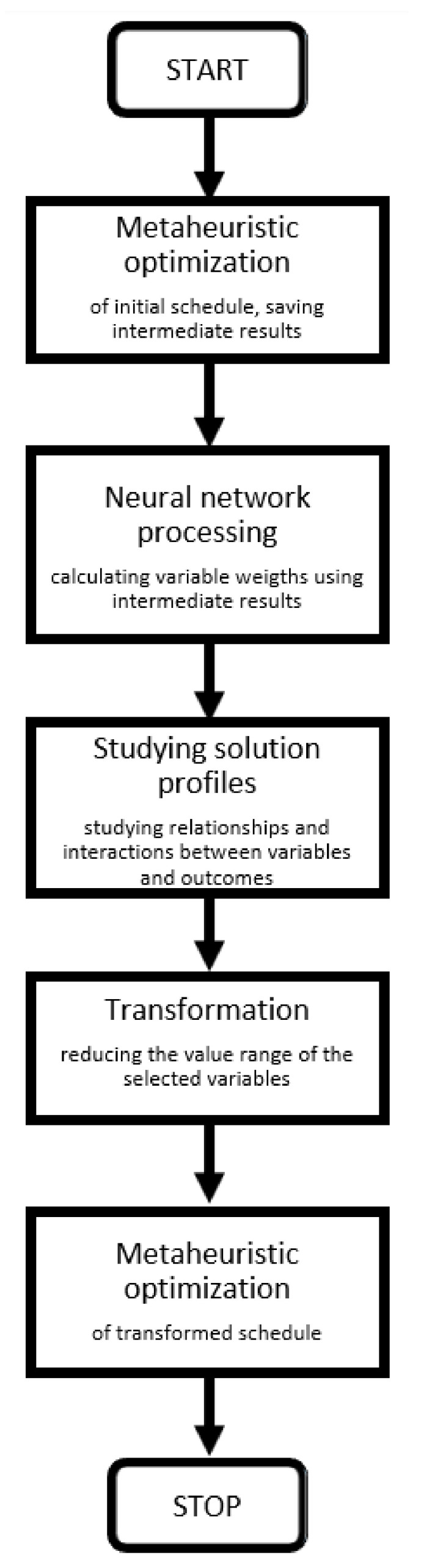
Block diagram of the AMTANN procedure.

The procedure described in detail in [14] was modified by introducing artificial intelligence (AI). AMTANN (Approach for MRCPSP Transformation with the use of Artificial Neural Networks) procedure [29] was modified and implemented to improve obtained results. The modified procedure is presented in Figure 1. The AMTANN procedure is presented separately in Figure 2 while AMTANN principles are described in the author’s previous paper [29].

The procedure begins with the preparation of the original (initial) version of the project schedule. This version is based on the functional and operational plan of the project. Then alternative versions of the project are created. The possible use of different materials, the use of different technologies, etc., are distinguished. The value of individual variants is assessed. As a result, the schedule is updated with additional modes (multi-mode version).

In the next step, metaheuristic algorithms are used to search for maximum and minimum values of optimized parameters. The constraints are not considered at this stage (UPS—Unconstrained Project Scheduling). The solutions found help to build a mathematical model and carry out the AMTANN analysis (Figure 2).

Finally, the results are assessed. If several acceptable solutions are obtained for different $w_{i}$ weight configurations, one of them should be selected. First of all, it should be checked whether some solutions are dominated by others. Only Pareto optimal solutions are eligible for the final selection. The final decision may be made by the decisionmaker arbitrarily or on the basis of one of the multi-criteria decision support methods, e.g., the AHP, TOPSIS, or ELECTRE method [37,38,39].

It may also happen that, despite finding acceptable schedules, the decisionmaker will not decide to implement the project, considering the obtained results to be insufficient. In this case, the presented approach can help to avoid losses of the enterprise related to the implementation of an inappropriate project.

The detailed procedure is presented on the real-life example in Section 3.

## 3. Results

### 3.1. Case Study

#### 3.1.1. Basic Information

The subject of the case study is a public utility building with two underground and seven above-ground stories, located at Domaniewska street in Warsaw, Poland. The object is described in detail in [40]. It is an office building (with commercial premises) about 30 m high. The analyzed building consists of two independent parts separated by a fire wall, and their only connection is in the underground garages. The basic parameters of the planned facility are presented in Table 1.

**Figure 3:**
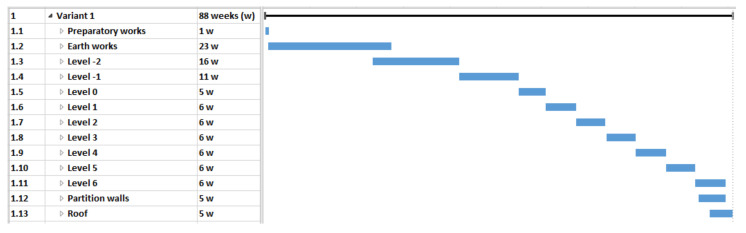
Case study—Variant 1 schedule—a pictorial screenshot.

**Figure 4:**
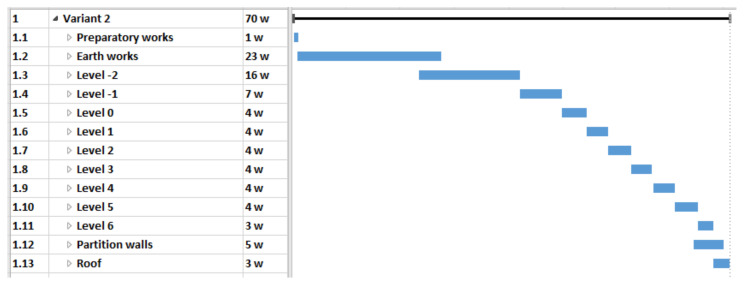
Case study—Variant 2 schedule—a pictorial screenshot.

**Figure 5:**
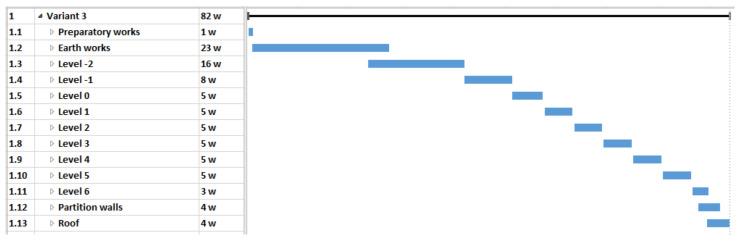
Case study—Variant 3 schedule—a pictorial screenshot.

In this case, the analysis covered the construction of the building in three different material variants. With the use of different materials, it was necessary to use a specific technology. The selection of materials also influenced the duration and cost of the project. The three original timetables for each option are as follows:

- variant 1 (V1)—reinforced concrete structure made of steel and concrete materials on the construction site (Figure 3),
- variant 2 (V2)—main structural elements in the prefabricated elements technology (Figure 4),
- variant 3 (V3)—mixed technology with the ceiling which consists of beams with a spatial truss and blocks made of light aggregate concrete (after laying the beams and blocks, the ceiling is flooded with concrete) (Figure 5).

#### 3.1.2. Value Analysis

Based on the original schedules and descriptions of individual variants, including materials [40], a table of variants was prepared with a short description of the assessed methods (Table 2). The duration of individual activities and the relationship between tasks were introduced on the basis of a previously prepared study [40]. In this example, the project manager considers the constraints of renewable resources (Z1: workers, Z2: concrete pumps, and Z3: cranes) and non-renewable resources (costs related to the implementation of individual activities, including material costs, which were calculated in the study [40]). The values of *V_i_* were determined on the basis of the value profile table (the evaluation of the values is presented below, using the example of the item “partition walls”). Table 3 shows the assessment of the various variants.

In the given example, some of the works at the beginning of construction are the same for all variants; their value has been assessed as equivalent and amounts to 1.0. Three material/technological variants described above were considered, while it was assumed that for economic and organizational reasons, the concept of the entire facility should be consistent, therefore for most works: level -1 (excluding the entry ramp) to level 6 (with a flat roof) a common/total value analysis was done. Each of the activities, depending on the selected design variant, received the same value within the corresponding variant. A separate analysis was performed only for three variants of the partition walls because this activity does not depend on the construction variant. The table of the value profile along with the significance of individual criteria assessment is presented below on the example of the partition wall (Table 4).

Based on the opinion of the expert team, after normalization, a vector of weights was obtained for the individual factors of creating the value-**Q** (Table 5).

After normalization, a normalized evaluation matrix with scores is obtained, as presented in Table 6.

A normalized **V** rating matrix is calculated, considering the importance of individual value-creating factors (Table 7).

The final scores for the individual variants (material solutions) are obtained by way of summation and standardization, and are presented in Table 8.

#### 3.1.3. Project Update

After the analysis, the calculated **V** values were entered into the schedules, and the relationships between tasks were updated, at the same time introducing the possibility of delaying activities, allowing for optimization of project parameters, and considering resource constraints and material solutions. The schedule also includes data on the contractual period, 130 weeks, and the deadline, 150 weeks. Indirect costs are also included.

#### 3.1.4. UPS Optimization

In the next step, a metaheuristic algorithm was used (the case study was calculated using OptQuest^®^ Engine, OptTek Systems, Inc.’s) to calculate maximum and minimum values of *NPV*, *CF*, and *V*: *NPV_max_*, *NPV_min_*, *CF_max_*, *CF_min_, V_max_,* and *V_min_*. The cash flow calculated in this example considered only the flows starting from the 10th month of construction because the work carried out in the first 9 months of the construction period was the same for all variants. It was assumed that the decisionmaker wants to optimize the cash flow during the construction of the above-ground part of the facility. The results are presented in Table 9. The analyzed variables were variants of materials used/works execution (three possible options for the structure and three for partition walls) and activity delays (zero to eight weeks depending on the activity). Such delays can help spread the cash flow caused by material orders or employee payments over time.

#### 3.1.5. MRCPS Optimization and Materials/Technology Selection

Penalties for exceeding the directive deadline (EUR 50 000 for a week of delay) were introduced into the computer model. Additionally, resource limitations were introduced: construction workers (64 workers), concrete pumps (5 pumps), cranes (2 cranes). The introduced limitations made the original three variants of the schedule unacceptable (they did not meet the imposed resource availability constraints). MRCPS optimization was performed for the ten sets of weights shown in Table 10.

The best results for each set of weights were recorded for later comparison with the results obtained by the AMTANN procedure. These results, along with random suboptimal solutions, were used as a sample for learning, validating, and testing the artificial neural network (2000 records in total). In the described case AMTANN was used to reduce the range of the variables.

**Figure 6:**
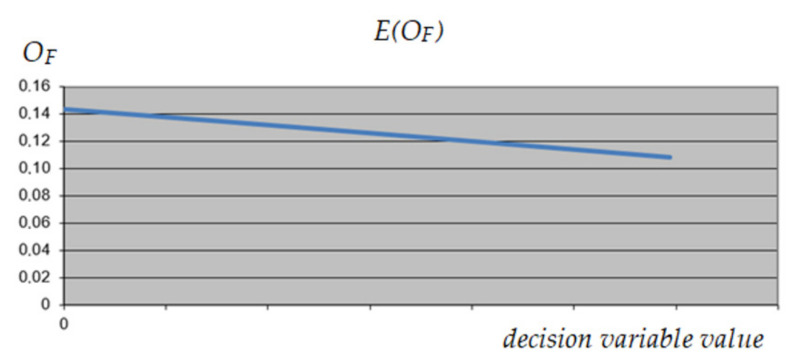
The expected value of *O_F_* depending on the value of the analyzed decision variable (activity 13)—maximum values of other variables.

**Figure 7:**
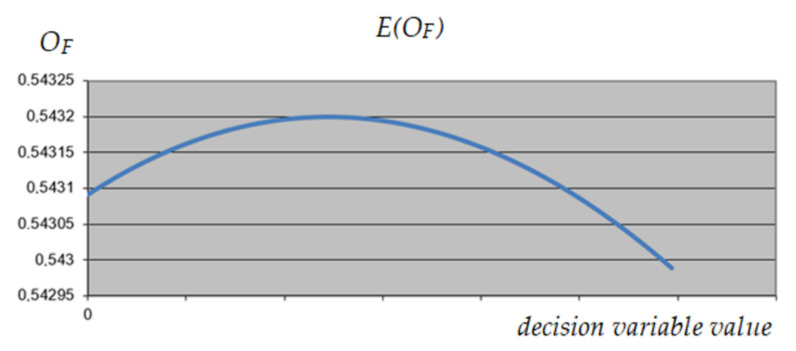
The expected value of *O_F_* depending on the value of the analyzed decision variable (activity 13)—minimum values of other variables.

**Figure 8:**
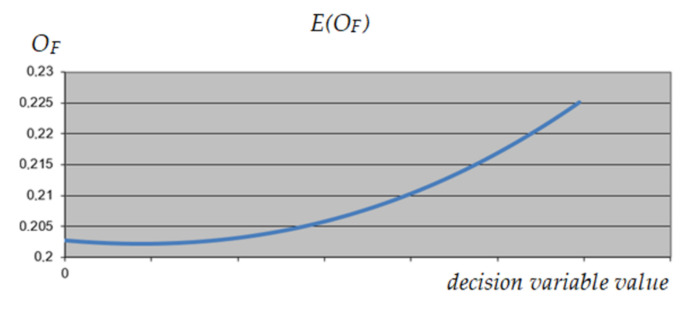
The expected value of *O_F_* depending on the value of the analyzed decision variable (activity 13)—intermediate values of other variables.

The method of selecting the reduced variables is presented in the example of activity 13—non-reduced variable (Level 0: Walls 2) and the construction variant—reduced variable (variable 1). After processing the neural network and establishing weights for each variable, solution profiles were examined to establish relationships between predictors (variables) and outcomes (output) and interactions between the predictors. For the constant (minimum, intermediate, and maximum) values of the predictors, the behavior of each of the variables in relation to the predicted result was checked. The profile of the analyzed variable, and possible delay of activity no. 13 in three variants is shown in Figure 6, Figure 7 and Figure 8.

Due to the lack of consistency of the profiles, it was decided not to reduce the value range of the variable corresponding to activity no. 13.

**Figure 9:**
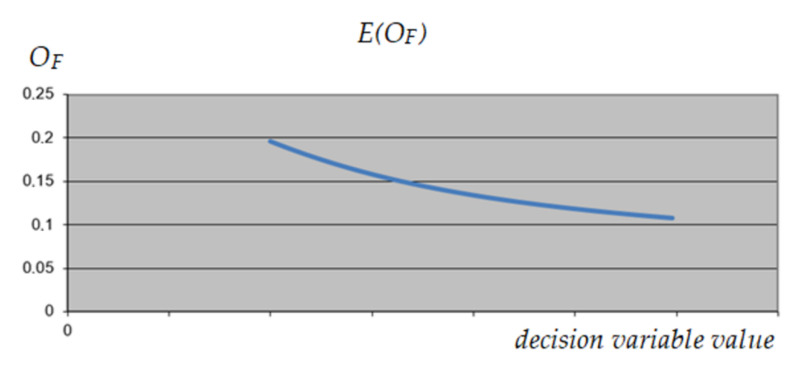
The expected value of *O_F_* depending on the value of the analyzed decision variable (variable 1)—maximum values of other variables.

**Figure 10:**
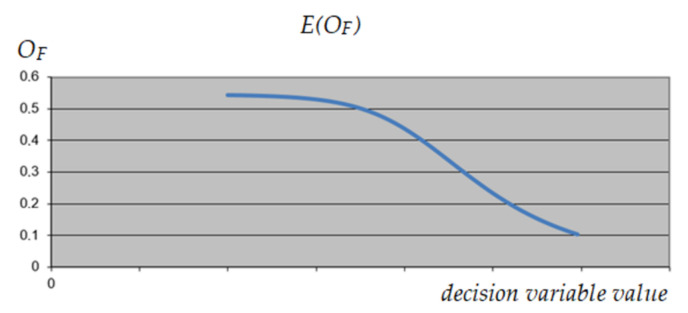
The expected value of *O_F_* depending on the value of the analyzed decision variable (variable 1)—minimum values of other variables.

**Figure 11:**
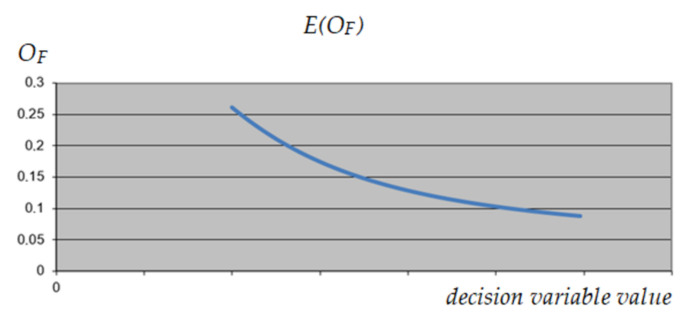
The expected value of *O_F_* depending on the value of the analyzed decision variable (variable 1)—intermediate values of other variables.

A similar analysis was performed on variable 1, corresponding to the selection of the construction variant of the object. As can be seen in Figure 9, Figure 10 and Figure 11, this variable has the same impact on the expected result, regardless of the value of the other variables, which qualifies it to reduce its range. As a result, it was decided to exclude variant 3 from further calculations.

**Figure 12:**
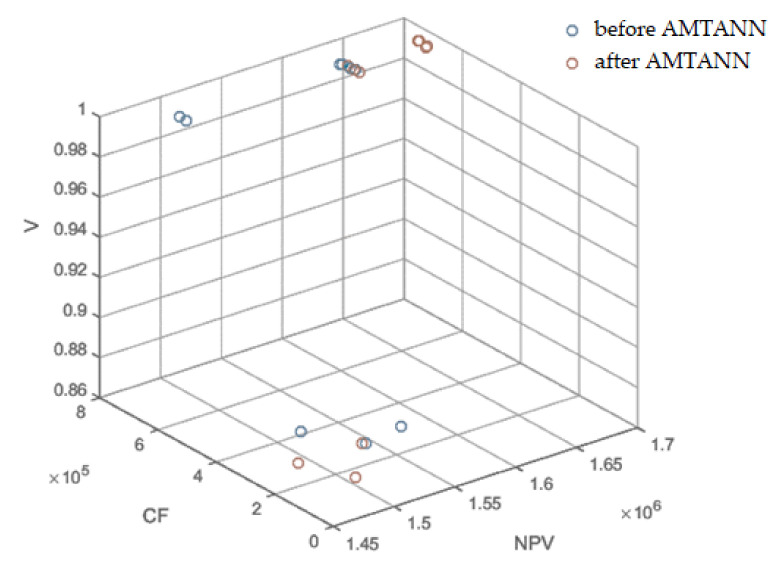
Results before and after the application of AMTANN for various configurations of weights of the objective function (NPV, CF and V)—3D view.

**Figure A1:**
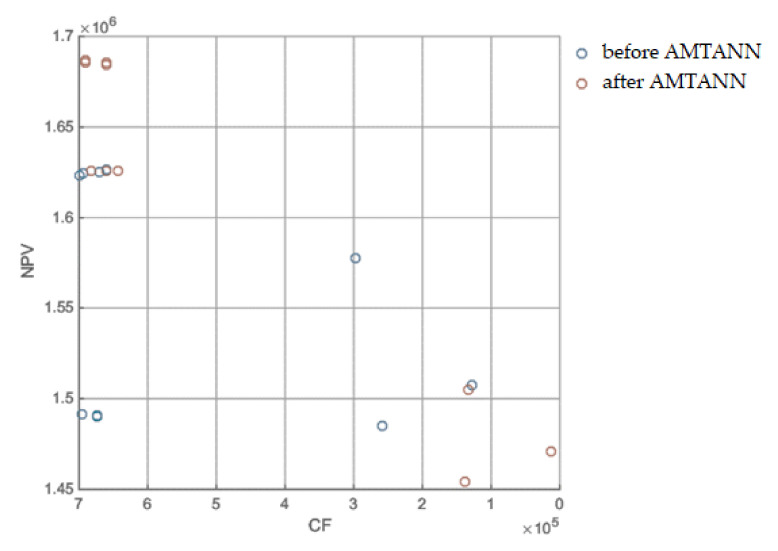
Results before and after the application of AMTANN for various configurations of weights of the objective function (NPV, CF, and V)—2D view: NPV and CF.

**Figure A2:**
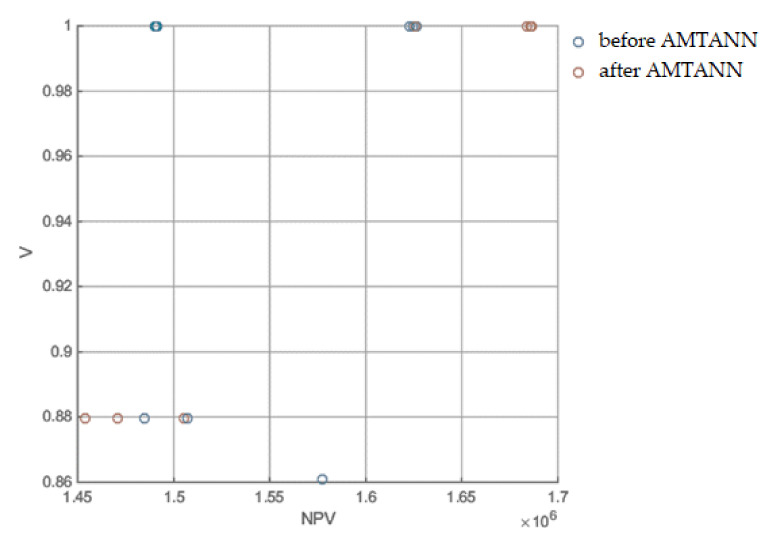
Results before and after the application of AMTANN for various configurations of weights of the objective function (NPV, CF, and V)—2D view: NPV and V.

**Figure A3:**
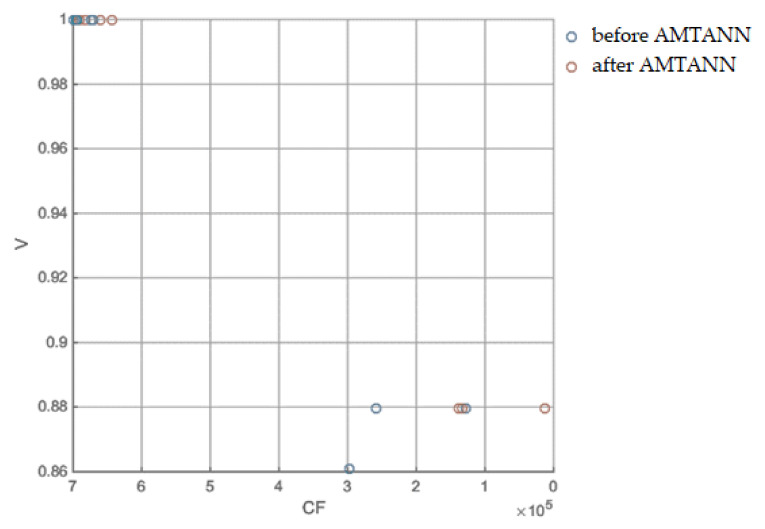
Results before and after the application of AMTANN for various configurations of weights of the objective function (NPV, CF, and V)—2D view: CF and V.

As a result of the procedure, the range of 13–35 variables was reduced. This procedure reduced the solution space significantly (by about 2∙10^24^ possible variants). The results before and after the application of AMTANN are presented below in Table 11 and Figure 12 (additional views are available in the Appendix A: Figure A1, Figure A2 and Figure A3).

In the individual columns of Table 11, the corresponding results (the same weights of the objective function) achieved better values after using the AMTANN procedure. As shown in Figure 12 and Appendix A, the results after using artificial neural networks showed less randomness and were generally better (greater average distance from the origin of the coordinate system and greater values of the objective function within the same sets of weights). Importantly, AMTANN assumes the preservation of the original results to confront them with the final results at the later stage, thanks to which some solutions belonging to the Pareto front are not lost.

#### 3.1.6. Variant Selection

**Figure 13:**
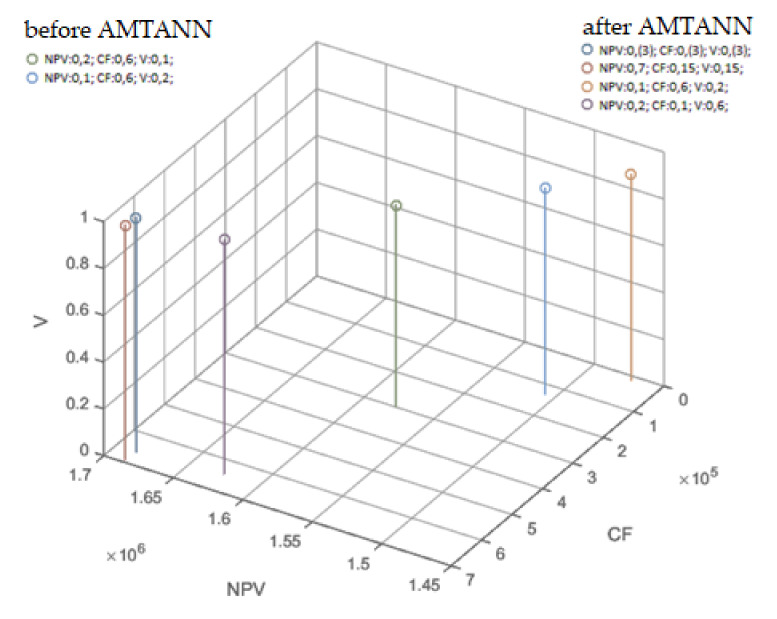
Solutions belonging to the Pareto front—3D view.

**Figure A4:**
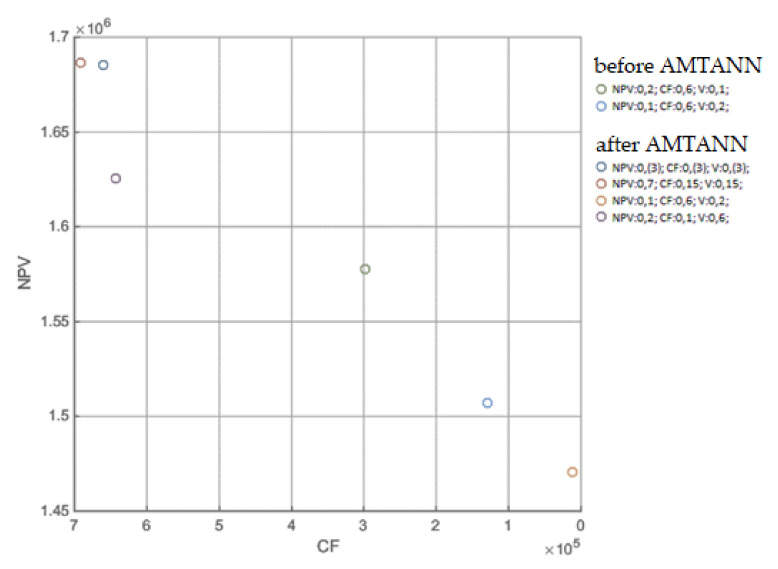
Solutions belonging to the Pareto front—2D view: NPV and CF.

**Figure A5:**
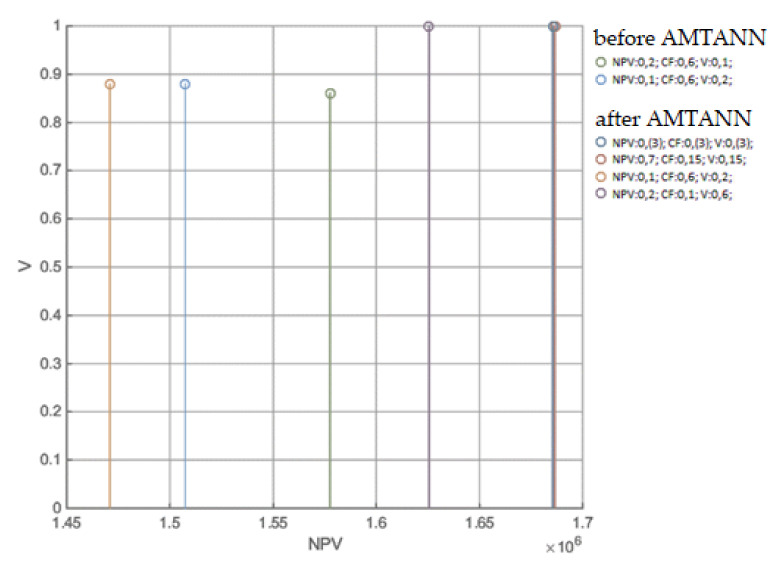
Solutions belonging to the Pareto front—2D view: NPV and V.

**Figure A6:**
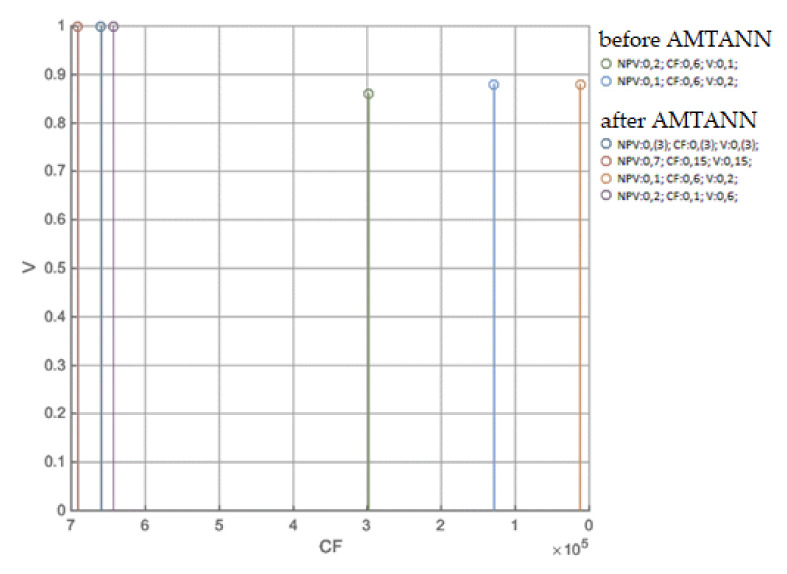
Solutions belonging to the Pareto front—2D view: V and CF.

Solutions belonging to the Pareto set (not dominated by any others) are presented in Figure 13 (in Appendix B, projections of points on the NPV, CF plane have been added to improve the legibility of the drawings—Figure A4, Figure A5 and Figure A6). Only these solutions were considered when selecting the variant of the final project. An alternative decision could have been to reject the project entirely. One of the multi-criteria decision-making methods (some presented here [41]) can be used in the final selection.

**Figure 14:**
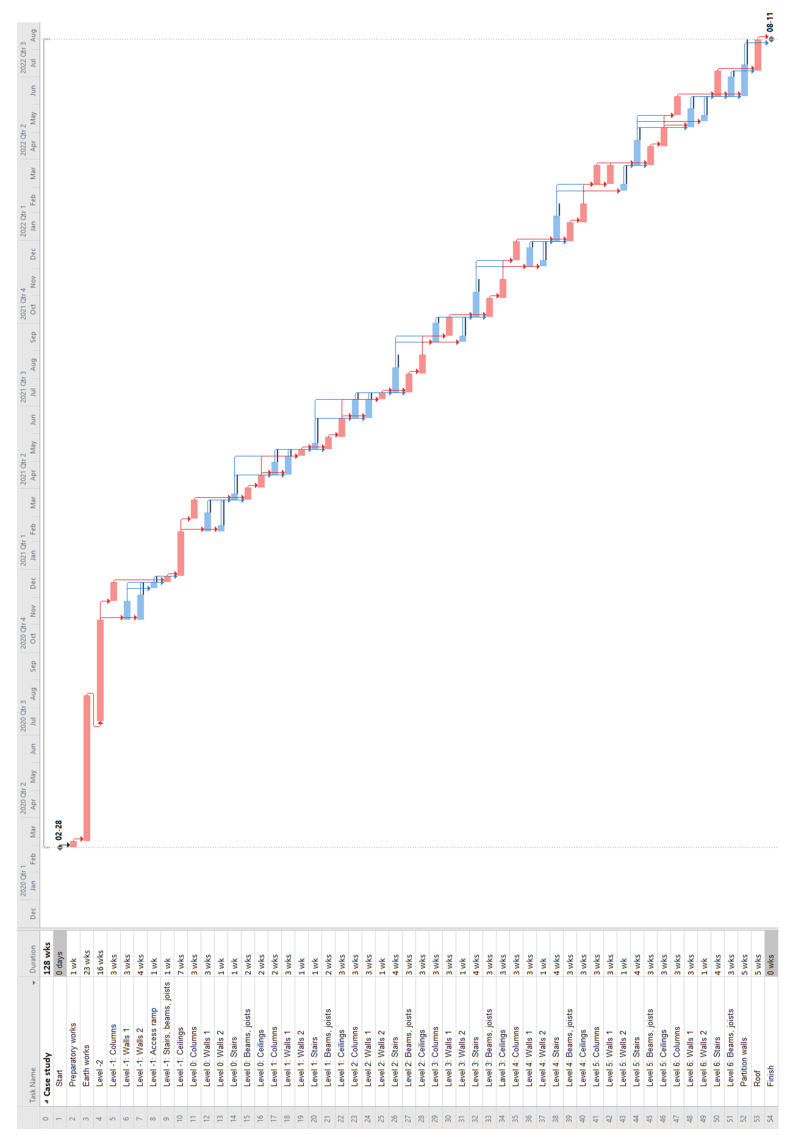
Case study—final schedule—illustrative screenshot.

In the analyzed case, the decisionmaker decided to choose the NPV variant: 0.7; CF: 0.15; and V: 0.15 from the AMTANN procedure; it has the highest possible V value and the highest NPV value among the options considered. An illustrative schedule of the selected variant is presented in Figure 14. Selected materials’ variants and delays’ values (final variable values) are presented in Table 12.

## 4. Discussion

The example presented above shows the effectiveness of the use of metaheuristic algorithms when selecting materials and technologies for a construction project. Moreover, according to the data in Table 11, these results can be further improved using artificial intelligence tools. Importantly, the presented methodology does not impose the only correct solution. Instead, it gives managers the option to choose from among the many advantageous solutions that make up the Pareto front (Figure 13). The summary of results for the final selection of weights is presented in Table 13.

Thanks to the use of metaheuristic optimization, a significant improvement in the results were achieved. Objective function value improved by 51.01% (in case of variant 2)–499.61% (variant 3). Moreover, thanks to the use of AMTANN, a further improvement of 12.43% was achieved (a total improvement over the initial options: 69.79–574.16%). Not only was there a significant improvement in the NPV parameter, which was dominant in this case. The parameter V, which is crucial from the point of view of durability and serviceability of the object, was also improved.

**Figure 15:**
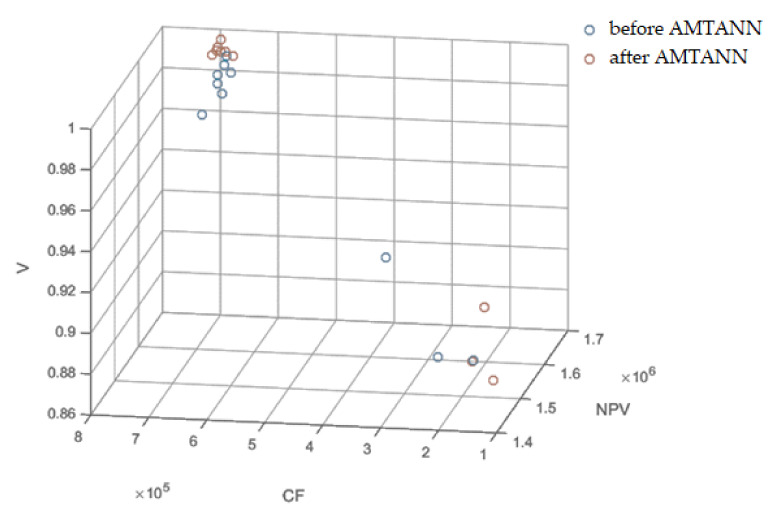
Average results before and after the application of AMTANN for different configurations of weights of the objective function (NPV, CF and V)—3D view.

**Figure A7:**
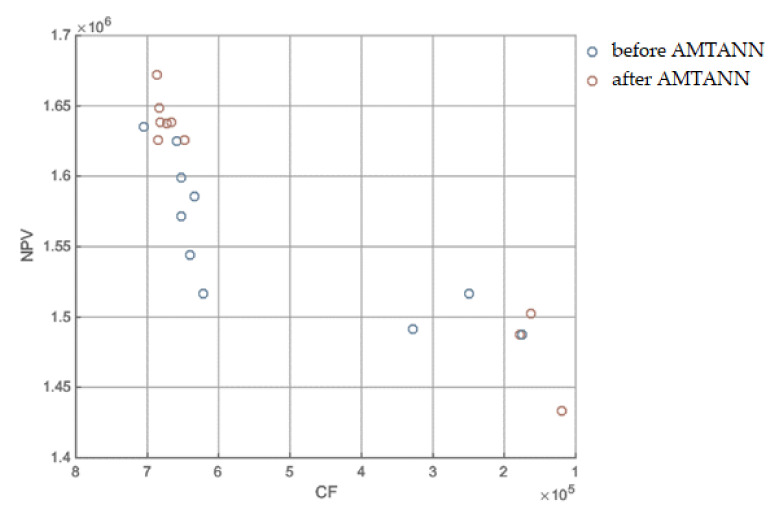
Average results before and after the application of AMTANN for different configurations of weights of the objective function (NPV, CF, and V)—2D view: NPV and CF.

**Figure A8:**
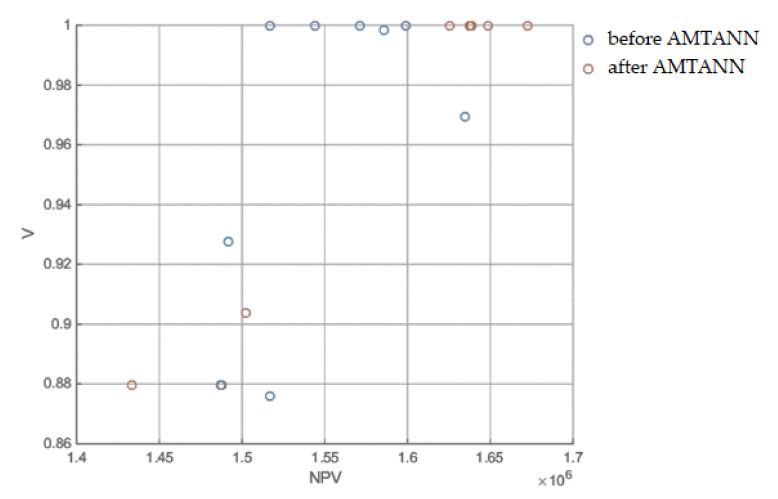
Average results before and after the application of AMTANN for different configurations of weights of the objective function (NPV, CF, and V)—2D view: NPV and V.

**Figure A9:**
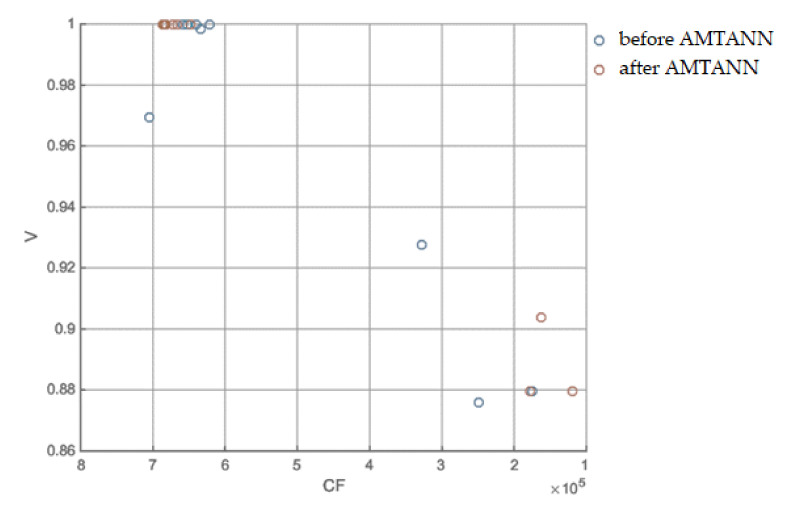
Average results before and after the application of AMTANN for different configurations of weights of the objective function (NPV, CF, and V)—2D view: V and CF.

To confirm the effectiveness of AMTANN, additional tests (calculations) were carried out for the example studied in this article. The mean results are shown in Figure 15 and in Appendix C (Figure A7, Figure A8 and Figure A9). Mean results after the application of ANN are characterized by higher values of *O_F_* and parameters *NPV*, *V*, and *CF*.

The presented method is so flexible that it can be used for projects of various sizes. So far, the author has studied single-family house-sized cases as well as multi-unit housing estates and commercial buildings. However, the method requires careful model building, which means that an experienced manager must be employed. However, this is now the standard for major projects.

Based on the conducted research and analyses, the following conclusions can be drawn:

1. It is possible to improve the functionality/usability of the facility by using appropriate materials and technological solutions.
2. It is possible to obtain a reliable assessment result and to select the variant of the undertaking most adequate to the formulated expectations of the decisionmaker.
3. It is possible to optimize the construction schedule by considering the economic and utility value of a construction project with the use of artificial intelligence tools.
4. Artificial neural networks can be effectively used to support the metaheuristic algorithm to improve project outcomes.

Moreover, the approach proposed by the author is structured in such a way that it can use various tools. In the future, the author plans to test and compare various artificial intelligence tools and optimization algorithms.

## 5. Conclusions

The proposed procedure allowed for the selection of the best available material/technological solution from the point of view of the decisionmaker. The use of AMTANN made it possible to find potential solutions better than those obtained using only the metaheuristic algorithm.

In the tests so far, improvement has been achieved in the majority of cases. Importantly, AMTANN retains the results from the original optimization, so even if the original results are not improved, the user retains the best results obtained during initial metaheuristic optimization. The proposed approach comprehensively reflects the complexity of construction processes. At the same time, it allows users to be flexible and adjust the tested parameters to their own needs. Thanks to the appropriate selection of material and technological solutions, the analyzed projects can achieve better economic results.

In the future, further development of the method is planned, including the use of other artificial intelligence tools.

## Figures and Tables

**Table 1:** Basic parameters of the office building.

ID	Data	Units	Value
1	Number of underground stories	-	2
2	Number of above-ground stories	-	7
3	Ground floor level	m above water level	24.5
**4**	**Total area**	**m^2^**	**44,875.67**
4.a	Underground area	m^2^	14,426.03
4.b	Above-ground area	m^2^	30,449.64
**5**	**Usable area**	**m^2^**	**36,784.17**
5.a	Office area	m^2^	22,445.40
5.b	Service premises area (ground floor)	m^2^	1,042.59
5.c	Auxiliary area	m^2^	1,483.98
5.d	Garage area	m^2^	12,051.53
6	Traffic area	m^2^	2454.82
**7**	**Cubature**	**m^3^**	**169,124.90**
7.a	Underground volume	m^3^	55,251.68
7.b	Above-ground volume	m^3^	113,873.30
8	Approximate number of employees	-	2556
**9**	**Parking spaces**	**unit**	**431**
9.a	In the garage	unit	394
9.b	Outside of the building	unit	41
9.c	Number of parking spaces per 1000 m^2^ of service area	-	25
9.d	Number of parking spaces per 1000 m^2^ of office area	-	18

**Table 2:** Description of materials’ variants—case study.

Task		Variant 1	Variant 2	Variant 3
ID	Name	Description	Description	Description
-	-	Monolithic construction	Prefabricated technology	Mixed technology with the use of light aggregate concrete blocks
1	Start	-	-	-
2	Preparatory works	Site fencing, tree clearing, temporary road laying, container assembly (the same for all variants)		
3	Earth works	Removal of plant soil, diaphragm walls, excavations, ceiling trim of level -2, temporary columns, excavation of level -2 (the same for all variants)		
4	Level -2	Lean concrete under the bottom slab, bottom slab, reinforced concrete columns, reinforced concrete walls, entry ramp, reinforced concrete stairs (the same for all variants)		
5	Level -1: Columns	Reinforced concrete columns formed in the system formwork.	Prefabricated columns	Prefabricated columns
6	Level -1: Walls 1	Reinforced concrete walls 25 cm thick (the same for all variants)		
7	Level -1: Walls 2	Reinforced concrete walls 20 cm thick (the same for all variants)		
8	Level -1: Access ramp	Reinforced concrete ramp 25 cm thick (the same for all variants)		
9	Level -1: Stairs, beams, joists	Reinforced concrete landings and flights of staircases with a slab thickness of 15 cm; reinforced concrete beams 50 cm × 30 cm; reinforcement degree: 110 kg/m^3^	Prefabricated stairs and beams	Prefabricated stairs and beams
10	Level -1: Ceilings	Monolithic reinforced concrete ceilings, 28 cm thick, with a degree of reinforcement of 95 kg/m^3^	Ceilings made of prefabricated hollow-core slabs	Thick-ribbed ceiling
11	Level 0: Columns	Reinforced concrete columns formed in the system formwork	Prefabricated columns	Prefabricated columns
12	Level 0: Walls 1	Reinforced concrete walls 25 cm thick (the same for all variants)		
13	Level 0: Walls 2	Reinforced concrete walls 20 cm thick (the same for all variants)		
14	Level 0: Stairs	Reinforced concrete landings and flights of staircases with a slab thickness of 15 cm	Prefabricated stairs	Reinforced concrete landings and flights of staircases with a slab thickness of 15 cm
15	Level 0: Beams, joists	Reinforced concrete beams 50 cm × 30 cm; reinforcement degree: 110 kg/m^3^	Prefabricated beams 600 cm × 30 cm × 30 cm	Prefabricated beams 600 cm × 30 cm × 30 cm
16	Level 0: Ceilings	Monolithic reinforced concrete ceilings, 28 cm thick, with a degree of reinforcement of 95 kg/m^3^	Ceilings made of prefabricated hollow-core slabs	Thick-ribbed ceiling
17	Level 1: Columns	Reinforced concrete columns formed in the system formwork	Prefabricated columns	Prefabricated columns
18	Level 1: Walls 1	Reinforced concrete walls 25 cm thick (the same for all variants)		
19	Level 1: Walls 2	Reinforced concrete walls 20 cm thick (the same for all variants)		
20	Level 1: Stairs	Reinforced concrete landings and flights of staircases with a slab thickness of 15 cm	Prefabricated stairs	Reinforced concrete landings and flights of staircases with a slab thickness of 15 cm
21	Level 1: Beams, joists	Reinforced concrete beams 50 cm × 30 cm. Reinforcement degree: 110 kg/m^3^	Prefabricated beams 600 cm × 30 cm × 30 cm	Prefabricated beams 600 cm × 30 cm × 30 cm
22	Level 1: Ceilings	Monolithic reinforced concrete ceilings, 28 cm thick, with a degree of reinforcement of 95 kg/m^3^	Ceilings made of prefabricated hollow-core slabs	Thick-ribbed ceiling
23	Level 2: Columns	Reinforced concrete columns formed in the system formwork	Prefabricated columns	Prefabricated columns
24	Level 2: Walls 1	Reinforced concrete walls 25 cm thick (the same for all variants)		
25	Level 2: Walls 2	Reinforced concrete walls 20 cm thick (the same for all variants)		
26	Level 2: Stairs	Reinforced concrete landings and flights of staircases with a slab thickness of 15 cm	Prefabricated stairs	Reinforced concrete landings and flights of staircases with a slab thickness of 15 cm
27	Level 2: Beams, joists	Reinforced concrete beams 50 cm × 30 cm; reinforcement degree: 110 kg/m^3^	Prefabricated beams 600 cm × 30 cm × 30 cm	Prefabricated beams 600 cm × 30 cm × 30 cm
28	Level 2: Ceilings	Monolithic reinforced concrete ceilings, 28 cm thick, with a degree of reinforcement of 95 kg/m^3^	Ceilings made of prefabricated hollow-core slabs	Thick-ribbed ceiling
29	Level 3: Columns	Reinforced concrete columns formed in the system formwork	Prefabricated columns	Prefabricated columns
30	Level 3: Walls 1	Reinforced concrete walls 25 cm thick (the same for all variants)		
31	Level 3: Walls 2	Reinforced concrete walls 20 cm thick (the same for all variants)		
32	Level 3: Stairs	Reinforced concrete landings and flights of staircases with a slab thickness of 15 cm	Prefabricated stairs	Reinforced concrete landings and flights of staircases with a slab thickness of 15 cm
33	Level 3: Beams, joists	Reinforced concrete beams 50 cm × 30 cm; reinforcement degree: 110 kg/m^3^	Prefabricated beams 600 cm × 30 cm × 30 cm	Prefabricated beams 600 cm × 30 cm × 30 cm
34	Level 3: Ceilings	Monolithic reinforced concrete ceilings, 28 cm thick, with a degree of reinforcement of 95 kg/m^3^	Ceilings made of prefabricated hollow-core slabs	Thick-ribbed ceiling
35	Level 4: Columns	Reinforced concrete columns formed in the system formwork	Prefabricated columns	Prefabricated columns
36	Level 4: Walls 1	Reinforced concrete walls 25 cm thick (the same for all variants)		
37	Level 4: Walls 2	Reinforced concrete walls 20 cm thick (the same for all variants)		
38	Level 4: Stairs	Reinforced concrete landings and flights of staircases with a slab thickness of 15 cm	Prefabricated stairs	Reinforced concrete landings and flights of staircases with a slab thickness of 15 cm
39	Level 4: Beams, joists	Reinforced concrete beams 50 cm × 30 cm; reinforcement degree: 110 kg/m^3^	Prefabricated beams 600 cm × 30 cm × 30 cm	Prefabricated beams 600 cm × 30 cm × 30 cm
40	Level 4: Ceilings	Monolithic reinforced concrete ceilings, 28 cm thick, with a degree of reinforcement of 95 kg/m^3^	Ceilings made of prefabricated hollow-core slabs	Thick-ribbed ceiling
41	Level 5: Columns	Reinforced concrete columns formed in the system formwork	Prefabricated columns	Prefabricated columns
42	Level 5: Walls 1	Reinforced concrete walls 25 cm thick (the same for all variants)		
43	Level 5: Walls 2	Reinforced concrete walls 20 cm thick (the same for all variants)		
44	Level 5: Stairs	Reinforced concrete landings and flights of staircases with a slab thickness of 15 cm	Prefabricated stairs	Reinforced concrete landings and flights of staircases with a slab thickness of 15 cm
45	Level 5: Beams, joists	Reinforced concrete beams 50 cm × 30 cm; reinforcement degree: 110 kg/m^3^.	Prefabricated beams 600 cm × 30 cm × 30 cm	Prefabricated beams 600 cm × 30 cm × 30 cm
46	Level 5: Ceilings	Monolithic reinforced concrete ceilings, 28 cm thick; reinforcement degree: 95 kg/m^3^	Ceilings made of prefabricated hollow-core slabs	Thick-ribbed ceiling
47	Level 6: Columns	Reinforced concrete columns formed in the system formwork	Prefabricated columns	Prefabricated columns
48	Level 6: Walls 1	Reinforced concrete walls 25 cm thick (the same for all variants)		
49	Level 6: Walls 2	Reinforced concrete walls 20 cm thick (the same for all variants)		
50	Level 6: Stairs	Reinforced concrete landings and flights of staircases with a slab thickness of 15 cm	Prefabricated stairs	Reinforced concrete landings and flights of staircases with a slab thickness of 15 cm
51	Level 6: Beams, joists	Reinforced concrete beams 50 cm × 30 cm; reinforcement degree: 110 kg/m^3^	Prefabricated beams 600 cm × 30 cm × 30 cm	Prefabricated beams 600 cm × 30 cm × 30 cm
52	Partition walls	NIDA plasterboards	SILKA sand-lime blocks	YTONG cellular concrete
53	Roof	Reinforced concrete roof, 28 cm thick; reinforcement degree: 95 kg/m^3^	Ceilings made of prefabricated hollow-core slabs	Thick-ribbed ceiling
54	Finish	-	-	-

**Table 3:** Assessment of variants materials/technology—case study.

	Variant 1						Variant 2						Variant 3					
ID	Cost [1000 EUR]	Duration [Weeks]	Value V	Z1	Z2	Z3	Cost [1000 EUR]	Duration [Weeks]	Value V	Z1	Z2	Z3	Cost [1000 EUR]	Duration [Weeks]	Value V	Z1	Z2	Z3
1	0.0	0	1.00	0	0	0	0.0	0	1.00	0	0	0	0.0	0	1.00	0	0	0
2	203.5	1	1.00	10	0	1	203.5	1	1.00	10	0	1	203.5	1	1.00	10	0	1
3	6714.0	23	1.00	24	0	0	6714.0	23	1.00	24	0	0	6714.0	23	1.00	24	0	0
4	5875.0	16	1.00	28	2	0	5875.0	16	1.00	28	2	0	5875.0	16	1.00	28	2	0
5	245.7	3	1.00	22	2	0	285.5	1	0.87	12	0	1	285.5	1	0.85	12	0	1
6	65.3	3	1.00	28	2	0	65.3	3	0.87	28	2	0	65.3	3	0.85	28	2	0
7	303.3	4	1.00	28	2	0	303.3	4	0.87	28	2	0	303.3	4	0.85	28	2	0
8	85.0	1	1.00	16	1	0	85.0	1	1.00	16	1	0	85.0	1	1.00	16	1	0
9	16.4	1	1.00	12	1	0	172.9	1	0.87	24	0	2	178.1	3	0.85	12	1	0
10	2068.3	7	1.00	40	2	0	902.6	2	0.87	12	0	2	1238.0	4	0.85	20	0	0
11	215.3	3	1.00	16	1	0	357.3	2	0.87	8	0	1	357.3	2	0.85	8	0	1
12	192.7	3	1.00	32	2	0	192.7	3	0.87	32	2	0	192.7	3	0.85	32	2	0
13	16.3	1	1.00	32	2	0	16.3	1	0.87	32	2	0	16.3	1	0.85	32	2	0
14	16.3	1	1.00	8	2	0	0 *	0	0.87	0	0	0	16.3	1	0.85	8	1	0
15	30.0	2	1.00	7	1	0	218.8	1	0.87	8	0	1	196.2	1	0.85	7	0	1
16	920.2	2	1.00	40	2	0	480.3	1	0.87	12	0	2	659.0	2	0.85	20	0	0
17	180.9	2	1.00	16	1	0	299.1	1	0.87	12	0	1	299.3	1	0.85	12	0	1
18	140.4	3	1.00	32	2	0	140.4	3	0.87	32	2	0	140.4	3	0.85	32	2	0
19	17.2	1	1.00	32	2	0	17.2	1	0.87	32	2	0	17.2	1	0.85	32	2	0
20	16.3	1	1.00	16	2	0	0 *	0	0.87	0	0	0	16.3	1	0.85	8	1	0
21	16.9	2	1.00	8	1	0	218.8	1	0.87	8	0	1	196.2	1	0.85	7	0	1
22	1109.6	3	1.00	40	2	0	573.8	1	0.87	12	0	2	787.4	2	0.85	20	0	0
23	199.8	3	1.00	16	1	0	339.8	1	0.87	12	0	1	340.1	1	0.85	12	0	1
24	144.0	3	1.00	32	2	0	144.0	3	0.87	32	2	0	144.0	3	0.85	32	2	0
25	17.2	1	1.00	32	2	0	17.2	1	0.87	32	2	0	17.2	1	0.85	32	2	0
26	16.3	4	1.00	16	2	0	0 *	0	0.87	0	0	0	16.3	1	0.85	8	1	0
27	16.9	3	1.00	8	1	0	218.8	1	0.87	8	0	1	196.2	1	0.85	7	0	1
28	1108.6	3	1.00	40	2	0	572.8	1	0.87	12	0	2	786.3	2	0.85	20	0	0
29	199.8	3	1.00	16	1	0	339.8	1	0.87	12	0	1	340.1	1	0.85	12	0	1
30	144.0	3	1.00	32	2	0	144.0	3	0.87	32	2	0	144.0	3	0.85	32	2	0
31	17.2	1	1.00	32	2	0	17.2	1	0.87	32	2	0	17.2	1	0.85	32	2	0
32	16.3	4	1.00	16	2	0	0 *	0	0.87	0	0	0	16.3	1	0.85	8	1	0
33	16.9	3	1.00	8	1	0	218.8	1	0.87	8	0	1	196.2	1	0.85	7	0	1
34	1104.8	3	1.00	40	2	0	572.8	1	0.87	12	0	2	786.3	2	0.85	20	0	0
35	199.8	3	1.00	16	1	0	339.8	1	0.87	12	0	1	340.1	1	0.85	12	0	1
36	144.0	3	1.00	32	2	0	144.0	3	0.87	32	2	0	144.0	3	0.85	32	2	0
37	17.2	1	1.00	32	2	0	17.2	1	0.87	32	2	0	17.2	1	0.85	32	2	0
38	16.3	4	1.00	16	2	0	0 *	0	0.87	0	0	0	16.3	1	0.85	8	1	0
39	16.9	3	1.00	8	1	0	218.8	1	0.87	8	0	1	196.2	1	0.85	7	0	1
40	1104.8	3	1.00	40	2	0	572.8	1	0.87	12	0	2	786.3	2	0.85	20	0	0
41	199.8	3	1.00	16	1	0	339.8	1	0.87	12	0	1	340.1	1	0.85	12	0	1
42	144.0	3	1.00	32	2	0	144.0	3	0.87	32	2	0	144.0	3	0.85	32	2	0
43	17.2	1	1.00	32	2	0	17.2	1	0.87	32	2	0	17.2	1	0.85	32	2	0
44	16.3	4	1.00	16	2	0	0 *	0	0.87	0	0	0	16.3	1	0.85	8	1	0
45	16.9	3	1.00	8	1	0	218.8	1	0.87	8	0	1	196.2	1	0.85	7	0	1
46	1104.8	3	1.00	40	2	0	572.8	1	0.87	12	0	2	786.3	2	0.85	20	0	0
47	200.1	3	1.00	16	1	0	339.8	1	0.87	12	0	1	340.1	1	0.85	12	0	1
48	144.8	3	1.00	32	2	0	144.8	3	0.87	32	2	0	144.8	3	0.85	32	2	0
49	17.3	1	1.00	32	2	0	17.3	1	0.87	32	2	0	17.3	1	0.85	32	2	0
50	16.3	4	1.00	16	2	0	0 *	0	0.87	0	0	0	16.3	1	0.85	8	1	0
51	65.4	3	1.00	8	1	0	218.8	1	0.87	8	0	0	196.2	1	0.85	7	0	1
52	890.4	5	0.62	32	0	0	495.8	5	1.00	32	0	0	416.9	4	0.99	32	0	0
53	1349.1	5	1.00	32	2	0	716.5	3	0.87	32	2	0	930.2	4	0.85	32	2	0
54	0.0	0	1.00	0	0	0	0.0	0	1.00	0	0	0	0.0	0	1.00	0	0	0

* In variant 2, the stairs are made together with beams and joists as part of the activities: Beams, joists.

**Table 4:** Value profile table (evaluation matrix **P**)—case study—partition walls.

		Criteria Score	V1	V2	V3
1 Safety	1.1 Structural safety	0	-	-	-
	1.2 Fire safety	10	1	5	5
	1.3 Usage safety	0	-	-	-
2 Comfort	2.1 Acoustic comfort	6	5	4	2
	2.2 Visual comfort (lighting)	0	-	-	-
	2.3 Hygrothermal comfort	2	2	4	4
	2.4 Serviceability	2	5	3	4
3 Health	3.1 Air quality	0	-	-	-
	3.2 Water supply and other utilities	0	-	-	-
	3.3 Waste disposal	0	-	-	-
4 Durability	4.1 Durability	10	2	4	5
5 Sustainable development	5.1 Energy saving	0	-	-	-
	5.2 Greenhouse gas emissions	0	-	-	-
	5.3 Economics (running costs)	10	3	5	5
	5.4 Dismantling and utilization	2	5	4	3

**Table 5:** Illustrative representation of the vector of weights for individual value-creating factors—**Q**.

Criterion	Weight
1.1 Structural safety	0
1.2 Fire safety	0.238095
1.3 Usage safety	0
2.1 Acoustic comfort	0.142857
2.2 Visual comfort (lighting)	0
2.3 Hygrothermal comfort	0.047619
2.4 Serviceability	0.047619
3.1 Air quality	0
3.2 Water supply and other utilities	0
3.3 Waste disposal	0
4.1 Durability	0.238095
5.1 Energy saving	0
5.2 Greenhouse gas emissions	0
5.3 Economics (running costs)	0.238095
5.4 Dismantling and utilization	0.047619

**Table 6:** Normalized evaluation matrix $\bar{P}$.

	1.1	1.2	1.3	2.1	2.2	2.3	2.4	3.1	3.2	3.3	4.1	5.1	5.2	5.3	5.4
**V**1	0.577	0.140	0.577	0.745	0.577	0.333	0.707	0.577	0.577	0.577	0.298	0.577	0.577	0.391	0.707
**V**2	0.577	0.701	0.577	0.596	0.577	0.667	0.424	0.577	0.577	0.577	0.596	0.577	0.577	0.651	0.566
**V**3	0.577	0.701	0.577	0.298	0.577	0.667	0.566	0.577	0.577	0.577	0.745	0.577	0.577	0.651	0.424

**Table 7:** Assessment matrix **V**.

	1.1	1.2	1.3	2.1	2.2	2.3	2.4	3.1	3.2	3.3	4.1	5.1	5.2	5.3	5.4
**V**1	0	1.400	0	4.472	0	0.667	1.414	0	0	0	2.981	0	0	3.906	1.414
**V**2	0	7.001	0	3.578	0	1.333	0.849	0	0	0	5.963	0	0	6.509	1.131
**V**3	0	7.001	0	1.789	0	1.333	1.131	0	0	0	7.454	0	0	6.509	0.849

**Table 8:** Assessment of material variants for activity 52 of the schedule.

Variant	Score V
**V**1-NIDA	0.617
**V**2-SILKA	1.000
**V**3-YTONG	0.989

**Table 9:** Calculated extreme values of *NPV*, *CF,* and *V* (UPS optimization).

Indicator	Value
$NPV_{max}$	1,705,955 EUR
$NPV_{min}$	130,827 EUR
$CF_{max}$	1,961,197 EUR
$CF_{min}$	0 EUR
$V_{max}$	1.000
$V_{min}$	0.853

**Table 10:** Configurations of objective function’s weights—a case study.

$w_{1}$ (NPV)	0.3(3)	0.7	0.15	0.15	0.6	0.6	0.2	0.1	0.2	0.1
$w_{2}$ (CF)	0.3(3)	0.15	0.7	0.15	0.2	0.1	0.6	0.6	0.1	0.2
$w_{3}$ (V)	0.3(3)	0.15	0.15	0.7	0.1	0.2	0.1	0.2	0.6	0.6

**Table 11:** Results for various weight configurations before and after use of AMTANN.

$w_{1}$(*NPV*)	0.33	0.7	0.15	0.15	0.6	0.6	0.2	0.1	0.2	0.1
$w_{2}($*V*)	0.33	0.15	0.15	0.7	0.1	0.2	0.1	0.2	0.6	0.6
$w_{3}($*CF*)	0.33	0.15	0.7	0.15	0.2	0.1	0.6	0.6	0.1	0.2
After use of AMTANN										
*NPV_r_*	0.98702	0.98770	0.84019	0.94922	0.98604	0.98721	0.87249	0.85077	0.94899	0.94912
*V_r_*	1.00000	1.00000	0.17832	1.00000	1.00000	1.00000	0.17977	0.17832	1.00000	1.00000
*CF_r_*	0.33629	0.35232	0.07045	0.34795	0.33629	0.35232	0.06773	0.00628	0.32780	0.33629
O_F_	0.549693	0.788542	0.103465	0.790189	0.624369	0.757091	0.151836	0.116973	0.757018	0.627654
Duration [d]	128	128	100	132	128	128	112	104	132	132
NPV [EUR]	1,685,507	1,686,580	1,454,234	1,625,965	1,683,972	1,685,801	1,505,111	1,470,891	1,625,603	1,625,812
V	1	1	0.879577	1	1	1	0.879788	0.879577	1	1
CF [EUR]	659,527	690,964	138,159	682,408	659,527	690,964	132,835	12,318	642,875	659,527
Before use of AMTANN										
*NPV_r_*	0.94736	0.86367	0.85967	0.94819	0.94854	0.94959	0.91856	0.87390	0.86345	0.86316
*V_r_*	1.00000	1.00000	0.17832	1.00000	1.00000	1.00000	0.05026	0.17977	1.00000	1.00000
*CF_r_*	0.35684	0.35486	0.13182	0.35391	0.34203	0.33629	0.15205	0.06562	0.34349	0.34349
O_F_	0.529644	0.70134	0.063422	0.789142	0.600717	0.736126	0.097509	0.083971	0.738341	0.617618
Duration [d]	132	136	108	132	132	132	112	112	136	136
NPV [EUR]	1,623,042	1,491,217	1,484,922	1,624,346	1,624,897	1,626,556	1,577,672	1,507,331	1,490,873	1,490,416
V	1	1	0.879577	1	1	1	0.860808	0.879788	1	1
CF [EUR]	699,835	695,946	258,534	694,083	670,794	659,527	298,192	128,695	673,655	673,655

**Table 12:** Results for various weight configurations before and after use of AMTANN.

Variable No.	Variable Name	Selected Variant
Variables concerning materials and technological variants (execution modes)		
1	Construction material variant	1
2	Partition walls variant	2
Delay variables (values in weeks)		
3	5. Level -1: Columns	3
4	6. Level -1: Walls 1	0
5	7. Level -1: Walls 2	0
6	8. Level -1: Access ramp	2
7	11. Level 0: Columns	2
8	12. Level 0: Walls 1	0
9	13. Level 0: Walls 2	0
10	14. Level 0: Stairs	0
11	17. Level 1: Columns	0
12	18. Level 1: Walls 1	0
13	19. Level 1: Walls 2	3
14	20. Level 1: Stairs	0
15	23. Level 2: Columns	0
16	24. Level 2: Walls 1	0
17	25. Level 2: Walls 2	3
18	26. Level 2: Stairs	0
19	29. Level 3: Columns	2
20	30. Level 3: Walls 1	3
21	31. Level 3: Walls 2	2
22	32. Level 3: Stairs	0
23	35. Level 4: Columns	3
24	36. Level 4: Walls 1	2
25	37. Level 4: Walls 2	2
26	38. Level 4: Stairs	0
27	41. Level 5: Columns	3
28	42. Level 5: Walls 1	3
29	43. Level 5: Walls 2	2
30	46. Level 5: Stairs	0
31	47. Level 6: Columns	2
32	47. Level 6: Walls 1	0
33	49. Level 6: Walls 2	1
34	51. Level 6: Beams, joists (in variant 2, together with the stairs)	0
35	52. Partition walls	0

**Table 13:** The summary of results for the final selection of weights.

	Metaheuristic Optimization Results		Initial Solutions		
	After AMTANN	Before AMTANN	Variant 1	Variant 2	Variant 3
*O_F_*	0.789	0.701	0.386	0.464	0.117
NPV [EUR]	1,686,580	1,491,217	759,324	1,210,342	488,605
V	1.000	1.000	0.993	0.880	0.861
CF [EUR]	690,964	695,946	468,775	552,907	645,295

## Appendix A

Projections for Figure 12.

## Appendix B

Projections for Figure 13.

## Appendix C

Projections for Figure 15.
